# Development of a Biomarker-Based Scoring System Predicting Early Recurrence of Resectable Pancreatic Duct Adenocarcinoma

**DOI:** 10.1245/s10434-021-10866-6

**Published:** 2021-10-04

**Authors:** Keinosuke Ishido, Norihisa Kimura, Taiichi Wakiya, Hayato Nagase, Yutaro Hara, Taishu Kanda, Hiroaki Fujita, Kenichi Hakamada

**Affiliations:** Department of Gastroenterological Surgery, Hirosaki University Graduate School of Medicine, Hirosaki, Japan

**Keywords:** Pancreatic ductal adenocarcinoma, Resectable, Early recurrence, CA19-9, SPan-1

## Abstract

**Background:**

Resectable pancreatic ductal adenocarcinoma (R-PDAC) often recurs early after radical resection, which is associated with poor prognosis. Predicting early recurrence preoperatively is useful for determining the optimal treatment.

**Patients and methods:**

One hundred and seventy-eight patients diagnosed with R-PDAC on computed tomography (CT) imaging and undergoing radical resection at Hirosaki University Hospital from 2005 to 2019 were retrospectively analyzed. Patients with recurrence within 6 months after resection formed the early recurrence (ER) group, while other patients constituted the non-early recurrence (non-ER) group. Early recurrence prediction score (ERP score) was developed using preoperative parameters.

**Results:**

ER was observed in 45 patients (25.3%). The ER group had significantly higher preoperative CA19-9 (*p* = 0.03), serum SPan-1 (*p* = 0.006), and CT tumor diameter (*p* = 0.01) compared with the non-ER group. The receiver operating characteristic (ROC) curve analysis identified cutoff values for CA19-9 (133 U/mL), SPan-1 (78.2 U/mL), and preoperative tumor diameter (23 mm). When the parameter exceeded the cutoff level, 1 point was given, and the total score of the three factors was defined as the ERP score. The group with an ERP score of 3 had postoperative recurrence-free survival (RFS) of 5.5 months (95% CI 3.02–7.98). Multivariate analysis for ER-related perioperative and surgical factors identified ERP score of 3 [odds ratio (OR) 4.63 (95% CI 1.82–11.78), *p* = 0.0013] and R1 resection [OR 3.20 (95% CI 1.01–10.17), *p* = 0.049] as independent predictors of ER.

**Conclusions:**

For R-PDAC, ER could be predicted by the scoring system using preoperative serum CA19-9 and SPan-1 levels and CT tumor diameter, which may have great significance in identifying patients with poor prognoses and avoiding unnecessary surgery.

Pancreatic ductal adenocarcinoma (PDAC) is a refractory disease with very poor prognosis. It is currently the fourth most common cause of cancer-related mortality in the USA, and has a 5-year survival rate of less than 10%.^1^ Based on the current incidence of PDAC, it is expected to become the second leading cause of cancer-related deaths in Europe and the USA by 2030.^2,3^ Although radical resection is considered to be a curative treatment, the 5-year survival rate after curative resection is only 15–20%.^4,5^

When PDAC is diagnosed as resectable on computed tomography (CT), better survival, with 5-year survival of around 40%, can be expected by promptly introducing adjuvant chemotherapy after radical resection.^6–8^ However, distant metastases are often detected early after resection,^9,10^ and some patients have to discontinue adjuvant chemotherapy. Therefore, the question of how to control such a malignant behavior has become a big challenge, even for patients with resectable PDAC.^11^

Previous studies investigated factors related to the uncontrollable behavior of PDAC.^12,13^ In particular, serum CA19-9 level is known to correlate with postoperative prognosis, and its association with early postoperative recurrence has also attracted attention.^14,15^ Preoperative serum CA19-9 level has been reported to be an independent factor that determines early postoperative recurrence, and it has been perceived as an indicator of poor postoperative prognosis.^16–18^ Other studies addressed the association between tumor immunonutrient factors, such as neutrophil-to-lymphocyte ratio (NLR) and modified Glasgow Prognostic Score (mGPS), and early postoperative recurrence.^19,20^ The significance of neoadjuvant therapy (NAT) for such difficult-to-control PDACs has been discussed.^21,22^ The purpose of NAT is to achieve more reliable R0 resection and control latent distant metastasis preoperatively, which is expected to have a further prognostic effect on resectable PDAC.^23^ However, it has been also pointed out that the tumor may progress during the NAT period, and the surgical tolerance may decrease due to adverse events of NAT. A phase 2 randomized controlled trial comparing the contribution of perioperative FOFIRINOX and gemcitabine/nab-paclitaxel to survival in patients with R-PDAC was reported.^24,25^ According to the results, the 2-year survival rate, the primary endpoint, was 43% in the FOFIRINOX arm and 46.9% in the gemcitabine/nab-paclitaxel arm, with no significant difference between the groups. Furthermore, neither arm met the prespecified threshold of a 2-year overall survival rate of 58% based on historical data from adjuvant trials in R-PDAC. Therefore, this phase 2 trial did not have the expected outcome. Additionally, 75% of eligible patients were able to complete preoperative chemotherapy and undergo surgery, whereas the remaining patients were unable to undergo surgery because of disease progression or adverse events from preoperative chemotherapy. Thus, it is not well established whether NAT should be given to patients with resectable PDAC. It may be suggested that NAT is not appropriate for all cases of resectable PDAC. Hence, resectable PDAC should be further classified depending on the malignant potential of the tumor, and an appropriate treatment strategy should be chosen accordingly. In this context, pretreatment prediction of early recurrence is essential for treatment selection and improving the prognosis of resectable PDAC.

The aim of this study was to establish a prognostic scoring system based on preoperative biomarkers to identify early recurrence within 6 months after radical resection for resectable PDAC.

## Patients and Methods

### Ethics

This retrospective study was approved by the Ethics Committee of the Graduate School of Medicine, Hirosaki University. We obtained informed consent from all patients with regard to analyzing the data.

### Patients

From January 2005 to December 2019, 200 patients were diagnosed with resectable PDAC using multi-detector row computed tomography (MDCT) at Hirosaki University Hospital. Preoperative MDCT was performed in all patients, and contrast-enhanced magnetic resonance imaging (MRI) and positron emission tomography–computed tomography (PET–CT) were performed as ancillary diagnostic imaging. When the diagnostic images were not enough to diagnose with PDAC, endoscopic ultrasound-guided fine needle aspiration (EUS-FNA) was applied to confirm the diagnosis. Among these 200 patients, we excluded 22 patients (2 individuals in whom only exploratory laparotomy was performed due to distant metastases, 2 individuals in whom palliative surgery was performed, 7 patients in whom radical surgery could not be obtained, and 11 patients in whom preoperative chemotherapy was performed). Finally, 178 cases were analyzed. Among these 178 patients, 45 patients with recurrence occurring within 6 months after radical surgery were included in the early recurrence group (ER group), whereas 133 patients in whom recurrence did not occur within 6 months constituted the non-early recurrence group (non-ER group). We compared the clinicopathological parameters between the two groups.

### Definition of Resectable PDAC

Classification of resectability was performed in accordance with the National Comprehensive Cancer Network guidelines version 1 2020.^26^ Tumors meeting the following criteria were defined as resectable PDAC: (1) the tumor does not contact the major arteries (the celiac trunk, superior mesenteric artery, and common hepatic artery); (2) the tumor has no contact with the portal vein and superior mesenteric vein, or the contact is less than half of the circumference of the vessel wall, and it is not accompanied by venous contouring; (3) no distant metastasis was confirmed.

### Postoperative Complications

Postoperative complications were evaluated based on the classification reported by Clavien et al.^27^ Postoperative pancreatic fistula was defined as grade B and grade C based on the grading of the International Study Group of Pancreatic Fistula.^28^

### Pathological Evaluation

Pathological evaluation of surgical specimens was based on the tumor–node–metastasis (TNM) classification system of malignant tumors by the International Union Against Cancer (UICC, 8th edition).^29^ Evaluation of the excised margin was performed as follows: if there were no tumor cells on the pancreatic resection margin, nerve plexus dissection margin, portal vein dissection surface, posterior dissection surface, and bile duct dissection margin, it was evaluated as an R0 resection. In contrast, if the tumor cells were recognized on these margins, the resection was evaluated as an R1 resection. In all cases, rapid intraoperative pathological diagnosis of the pancreatic resection margin was conducted, and additional resection was performed when tumor cells were still present on the stump surface.

### Postoperative Adjuvant Chemotherapy

Postoperative adjuvant chemotherapy was introduced within 12 weeks after surgery and continued for 6 months. The chemotherapy regimen included gemcitabine (GEM) monotherapy, S-1 monotherapy, GEM and S-1 combination therapy, or GEM and nanoparticle albumin bound-paclitaxel combination therapy. No postoperative radiation was given in any of the patients. After the operation, blood examination including tumor markers (CA19-9, SPan-1, and DUPAN-2) every 4 weeks and CT examination every 3 months were performed to screen for postoperative recurrence. If the assessment of recurrence was uncertain, contrast-enhanced MRI or PET-CT was used as an adjunct method.

### Prediction Scoring System for Early Recurrence

Univariate analysis with preoperative factors was performed to identify the factors associated with early recurrence. For the identified factors, the cutoff values for predicting early recurrence were estimated using the ROC curve analysis. For the factors exceeding the cutoff value, 1 point was added. The total score of the identified factors was taken as the early recurrence prediction score (0 point, 46 cases; 1 point, 64 cases; 2 points, 37 cases; 3 points, 31 cases).

### Statistical Analysis

Categorical variables were compared between ER and non-ER groups with the chi-squared test or Fisher’s exact test. Variables with *p* < 0.05 were considered significant. Univariate and multivariate analyses of perioperative factors associated with early recurrence were conducted using logistic regression to identify independent factors related to early recurrence. The analysis of prognosis was based on the Kaplan–Meier curves. Log-rank test was used for comparison of progression-free survival and OS by early recurrence prediction scores, and *p* < 0.05 indicated a significant difference. Statistical evaluation was performed using IBM SPSS statistics version 25 for Windows.

## Results

### Patients’ Background

**Fig. 1:**
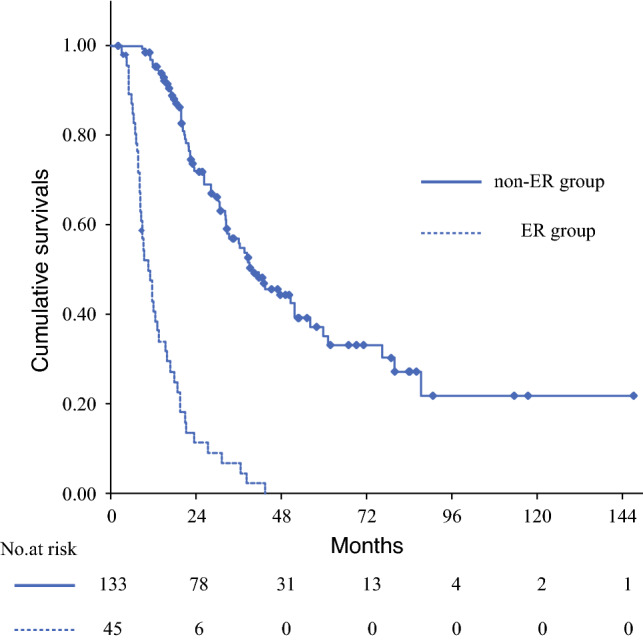
Comparison of survivals between ER and non-ER groups. On survival analysis by the Kaplan–Meier curve, the 3-year survival rate, 5-year survival rate, and median survival time of the early recurrence group (solid line) were 7.1%, 0.0%, and 10.9 months (95% CI 7.8–14.0), respectively, while the non-early recurrence group (dotted line) had 3-year survival rate, 5-year survival rate, and median survival time of 57.6%, 35.5%, and 40.0 months (95% CI 29.6–50.5), respectively. Log-rank test showed a significant difference in the survival curves between the two groups (*p* < 0.001)

The background information of 178 patients with resectable PDAC is presented in Table 1. The median age was 70 years, and the age range was 49–85 years. There were 86 women and 92 men. As comorbidities, diabetes mellitus was found in 55 cases (30.9%) and chronic pancreatitis in 7 cases (3.9%). The location of PDAC was most often in the head of the pancreas (118 cases), followed by the body and tail of the pancreas in 60 cases. The median values of preoperative serum carbohydrate antigen (CA)19-9, serum s-Pancreas-1 antigen (SPan-1), and Duke Pancreas-2 Antigen (DUPAN-2) were 69 U/mL [interquartile range (IQR) 20–217 U/mL], 51.3 U/mL (IQR 18.9–119.7 U/mL), and 99.5 U/mL (IQR 27.8–412.8 U/mL), respectively. The performed surgical procedures included pancreaticoduodenectomy, distal pancreatectomy, and total pancreatectomy in 108 cases (60.7%), 60 cases (33.7%), and 10 cases (5.6%), respectively. R0 and R1 resection were performed in 159 cases (89.3%) and 19 cases (10.7%), respectively. The median observation period was 22.3 months (8.0–147.2 months). The median overall survival was 30.6 months (95% CI 25.5–35.7), while the 5-year survival rate was 25.9%. The median recurrence-free survival (RFS) was 13.3 months (95% CI 10.7–15.9), and the 3-year RFS rate was 28.9%. Early recurrence was observed in 45 cases (25.2%). The sites of early recurrence included the liver, lungs, peritoneum, distant lymph nodes, and local areas after resection in 26 cases (57.8%), 2 cases (4.4%), 14 cases (31.3%), 10 cases (22.2%), and 12 cases (26.7%), respectively. Three-year survival rate, 5-year survival rate, and median survival time for the non-ER group were 57.6%, 35.5%, and 40.0 months (95% CT, 29.6–50.5), respectively, whereas those for the early recurrence group were 7.1%, 0.0%, and 10.9 months (95% CI 7.8–14.0), respectively. Significant differences in the OS were observed between the two groups (*p* < 0.001, Fig. 1).

**Table 1:** Patient characteristics

Parameter	
Number of patients	178
Age (years)	70 (49–85)
Gender (female/male)	86/92
BMI	22.3 (14.1–36.8)
ASA-PS (1/2/3/4<)	26/133/19/0
Diabetes mellitus	55 (30.9%)
Chronic pancreatitis	7 (3.9%)
Tumor location (Ph/Pbt)	118 / 60
Diameter of the pancreatic tumor on CT (mm)	25 (8–59)
Preoperative CA19-9 (U/mL)	69 (20–217)
Preoperative SPAN-1 (U/mL)	51.3 (18.9–119.7)
Preoperative DUPAN-2 (U/mL)	99.5 (27.8–412.8)
Operative procedures	
(PD/DP/TP)	108/60/10
Intraoperative transfusion	32 (18.0%)
Lymph node metastasis	108 (60.7%)
R0 resection rate	159 (89.3%)
Overall morbidity (Clavien–Dindo > II)	33 (18.5%)
90-day mortality	0 (0.0%)
Induction rate of adjuvant chemotherapy	142 (79.8%)
Median recurrence-free survival time (months)	13.3 (10.7–15.9)
Median survival time (months)	30.6 (25.5–35.7)
Postoperative recurrence within 6 months	45 (25.2%)
Recurrence site	
Liver	26 (57.8%)
Lung	2 (4.4%)
Peritoneum	14 (31.1%)
Distant lymph node	10 (22.2%)
Locoregional site	12 (26.7%)

*Age* age at diagnosis, *BMI* body mass index, *ASA-PS* American Society of Anesthesiologists physical status, *CA19-9* carbohydrate antigen 19-9, *Span-1* s-pancreas-1 antigen, *DUPAN-2* Duke pancreas-2 antigen, *PD* pancreaticoduodenectomy, *DP* distal pancreatectomy, *TP* total pancreatectomy

### Identification of Preoperative Factors Associated with Early Recurrence

We analyzed preoperative factors associated with early recurrence and found significantly higher levels of preoperative serum CA19-9 (1926.3 versus 292.1 U/mL; *p* = 0.03) and preoperative serum SPan-1 (673.1 versus 108.6 U/mL; *p* = 0.006) in the ER group. It was also revealed that diameter of the tumor on CT was significantly larger (39.4 versus 25.1 mm; *p* = 0.01). Serum DUPAN-2 levels tended to be higher in the ER group (1029.7 versus 526.8 U/mL; *p* = 0.07) in the ER group. Other preoperative factors such as treatment periods, comorbidities, preoperative nutritional indicators, tumor localization, and max value of standardized uptake value (SUVmax) on PET-CT did not show significant intergroup differences (Table 2). In our institute, gemcitabine/nab-paclitaxel and FOLFIRINOX have been available for PDAC treatment since 2015. Therefore, to assess the impact of effective chemotherapy, we divided the treatment period into early (2005–2014) and late periods (2015–2019) to assess the early recurrence rate. The early recurrence rates in the early and late periods were 30.8% (24/78) and 21.0% (21/100), respectively, with no significant difference between the periods (*p* = 0.14).

**Table 2:** Comparison of the predicting factors for early recurrence between ER and non-ER groups

Parameter	ER group	Non-ER group	*p*
Number of patients	45	133	
Age	69.3 ± 7.6	69.0 ± 7.7	0.75
Gender (female/male)	21/24	65/68	0.80
Period (2005–2014/2015–2019)	23/22	55/78	0.25
BMI	22.8 ± 2.9	22.5 ± 3.7	0.63
ASA score (1,2/3 ≤)	41/4	118/15	0.65
Diabetes mellitus	11 (24.4%)	44 (33.0%)	0.28
Chronic pancreatitis	1 (2.2%)	6 (4.5%)	0.49
Modified GPS (0/1/2)	37/5/3	102/26/5	0.34
NLR	2.8 ± 1.5	2.7 ± 1.9	0.78
PNI	44.9 ± 6.45	45.3 ± 6.2	0.70
Preoperative CA19-9 (U/mL)	1926.3 ± 8631.1	292.1 ± 963.1	0.03
Preoperative SPan-1 (U/mL)	673.1 ± 2270.1	108.6 ± 221.4	0.006
Preoperative DUPAN-2 (U/mL)	1029.7 ± 2939.8	526.8 ± 1247.7	0.07
SUVmax of the tumor on PET-CT	5.5 ± 2.3	5.5 ± 3.4	0.93
Tumor location (Ph/Pbt)	33/18	94/48	0.85
Diameter of the pancreatic tumor on CT	39.4 ± 10.1	25.1 ± 9.5	0.01

*ER* early recurrence, *Non-ER* no recurrence within 6 months after resection, *BMI* body mass index, *Age* age at diagnosis, *ASA-PS* American Society of Anesthesiologists physical status, *GPS* Glasgow Performance Status, *NLR* neutrophil-to-lymphocyte ratio, *PNI* prognostic nutritional index, *CA19-9* carbohydrate antigen 19-9, *Span-1* s-pancreas-1 antigen, *DUPAN-2* Duke pancreas-2 antigen, *SUVmax* max value of standardized uptake value, *PET* positron emission tomography, *CT* computed tomography, *Ph* pancreatic head, *Pbt* pancreatic body and tail

### Identification of Cutoff Values of Serum Tumor Markers and Tumor Diameter for Predicting Early Recurrence

ROC analysis of the factors associated with early recurrence presented in Table 2 was performed to set their cutoff values for early recurrence prediction (Table 3). The cutoff values of preoperative CA19-9, SPan-1, and CT-derived tumor diameter were 133 U/mL [area under curve (AUC) 0.688 (0.592–0.783); *p* < 0.001], 78.2 U/mL [AUC 0.721 (0.633–0.809); *p* < 0.001], and 23 mm [AUC 0.629 (0.535–0.723); *p* = 0.007], respectively. The cutoff values for preoperative serum DUPAN-2 and SUVmax on PET-CT were 126 U/mL [AUC 0.594 (0.499–0.670); *p* = 0.052] and 4.5 [AUC 0.522 (0.490–0.754); *p* = 0.07]. However, the two parameters could not be set as a significant cutoff values for predicting early recurrence.

**Table 3:** ROC curve analysis for predicting early recurrence

Parameters	Cutoff value	AUC	*p*
Preoperative CA19-9	133 U/mL	0.688 (0.592–0.783)	< 0.001
Preoperative SPan-1	78.2 U/mL	0.721 (0.633–0.809)	< 0.001
Preoperative DUPAN-2	126 U/mL	0.594 (0.499–0.670)	0.052
SUV max on PET-CT	4.5	0.522 (0.490–0.754)	0.07
Diameter of the tumor on CT	23.0 mm	0.629 (0.535–0.723)	0.007

*AUC* area under the curve, *CA19-9* carbohydrate antigen 19-9, *Span-1* s-pancreas-1 antigen, *DUPAN-2* Duke pancreas-2 antigen, *SUVmax* max value of standardized uptake value, *PET* positron emission tomography, *CT* computed tomography

### Stratification of Survival Time by Early Recurrence Prediction Score

**Fig. 2:**
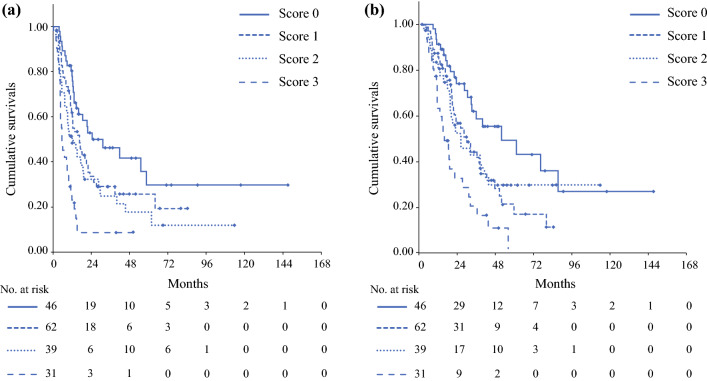
Stratification of survivals by the early recurrence prediction scores. Survival analysis using the Kaplan–Meier curve was performed stratified by early recurrence prediction scores. **A** Recurrence-free survival (mRFS) time for cases with scores of 0, 1, 2, and 3 were 24.4 months (95% CI 0.2–49.1), 16.5 months (95% CI 10.7–22.4), 10.2 months (95% CI 4.0–16.5), and 5.5 months (95% CI 3.0–8.0), respectively, showing a significant difference in the log-rank test (*p* < 0.001). **B** The median OS time of scores 0, 1, 2, and 3 was 51.8 months (95% CI 18.0–85.6), 32.3 months (95% CI 21.8–42.8), 23.1 months (95% CI 14.9–31.3), and 15.9 months (95% CI 10.4–21.4), respectively, also showing a significant difference in the log-rank test (*p* < 0.001)

The scoring system for predicting early recurrence was created using the cutoff values of preoperative serum CA19-9 (133 U/mL) and SPan-1 (78.2 U/mL), and preoperative CT-derived tumor diameter (23 mm). When the parameter exceeded the cutoff level, 1 point was given, and the total score of the three factors was defined as the early recurrence predictive score. Scores of 0, 1, 2, and 3 points were found in 46 cases, 62 cases, 39 cases, and 31 cases, respectively. The comparison of RFS and OS between the prediction scores showed that the median RFS time in cases with scores 0, 1, 2, and 3 was 24.4 months (95% CI −0.23–49.1), 16.5 months (95% CI 1.07–22.3), 12.1 (95% CI 5.56–18.7), and 5.5 months (95% CI 3.02–7.80), respectively. The median OS was 51.8 months (95% CI 18.0–85.6), 30.2 months (95% CI 20.5–40.0), 26.2 months (95% CI 12.0–40.4), and 15.9 months (95% CI 10.4–21.4) in cases with scores of 0, 1, 2, and 3, respectively. In the Kaplan–Meier survival analysis, both the RFS time and the OS time were significantly stratified by prediction scores (*p* < 0.001, Fig. 2).

### Univariate and Multivariate Analysis of Factors Predicting Early Recurrence

Univariate and multivariate analysis using the perioperative factors were performed to identify the independent factors associated with early recurrence (Table 4). On univariate analysis, early recurrence prediction score of 3 points (*p* < 0.001), postoperative complications (Clavien–Dindo > grade II, *p* = 0.01), pancreatic fistula (grades B and C, *p* = 0.04), lymphatic vessel invasion (*p* = 0.02), lymph node metastasis (*p* = 0.02), and vein invasion (*p* = 0.006) were associated with early recurrence. R1 resection (*p* = 0.05) and no postoperative adjuvant chemotherapy (*p* = 0.07) tended to be related with early recurrence; however, they were not significant. Furthermore, multivariate analysis showed that early recurrence prediction score of 3 [odds ratio (OR), 4.63; 95% CI 11.82–11.78, *p* = 0.0013] and R1 resection (OR 3.20; 95% CI 1.01–10.17, *p* = 0.049) were independently associated with recurrence within 6 months after curative resection for PDAC.

**Table 4:** Uni- and multivariate analysis of the predicting factors for early recurrence

Parameters	Univariate analysis			Multivariate analysis		
	ER group	Non-ER group	*p*	Odds ratio	95% CI	*p*
Number of patients	45	133				
Age (75 years <)	11 (24.4%)	32 (24.0%)	0.94			
Gender (male)	24 (53.3%)	68 (51.1%)	0.80			
BMI (25 <)	22.8 ± 2.9	22.5 ± 3.7	0.63			
ASA-PS (3 ≤)	4 (8.9%)	15 (11.3%)	0.65			
Diabetes mellitus	11 (24.4%)	44 (33.0%)	0.28			
Chronic pancreatitis	1 (2.2%)	6 (4.5%)	0.49			
Modified GPS (2)	3 (6.7%)	5 (3.8%)	0.45			
ER predictive score (3)	18 (40.0%)	14 (10.5%)	< 0.001	4.63	1.82–11.78	0.0013
Tumor location (Ph)	33 (73.3%)	94 (70.7%)	0.85			
Blood loss (1000 ml <)	20 (44.4%)	43 (32.3%)	0.052			
Operative time (360 min <)	16 (35.6%)	44 (33.1%)	0.88			
PV/SMV resection (+)	9 (20.0%)	21 (15.8%)	0.69			
Transfusion (+)	12 (26.7%)	32 (24.1%)	0.13			
PV/SMV invasion (+)	12 (26.7%)	25 (18.8%)	0.26			
Overall morbidity (Clavien–Dindo > II)	15 (33.3%)	19 (14.3%)	0.003	1.80	0.32–10.1	0.51
PF (Grade B, C)	11 (24.4%)	14 (10.5%)	0.018	2.60	0.37–18.02	0.33
Lymphatic invasion (+)	37 (82.2%)	81 (60.9%)	0.006	1.75	0.61–5.06	0.30
Venous invasion (+)	36 (80.0%)	85 (63.9%)	0.03	2.16	0.78–5.92	0.14
Neural invasion (+)	39 (86.7%)	103 (77.4%)	0.14			
Lymph node metastasis (+)	34 (75.6%)	74 (55.6%)	0.02	1.55	0.59–4.10	0.37
R1 resection	8 (17.8)	10 (7.5)	0.051	3.20	1.01–10.17	0.049
Adjuvant chemotherapy (−)	13 (28.9%)	22 (16.5%)	0.07	2.12	0.76–2.06	0.15

*ER* early recurrence, *Non-ER* no recurrence within 6 months after resection, *95% CI* 95% confidence interval, *Age* age at diagnosis, *BMI* body mass index, *ASA-PS* American Society of Anesthesiologists physical status, *GPS* Glasgow Performance Status, *Ph* pancreatic head, *PV* portal vein, *SMV* superior mesenteric vein, *PF* pancreatic fistula

### Recurrence Patterns in the Early Recurrence Group

The characteristics of the recurrence site in the two groups were examined (Table 5). It was shown that liver and peritoneal metastases were significantly more common in the ER than in the non-ER group (liver: 54.9% versus 47.1%, *p* < 0.001; peritoneum: 33.3% versus 12.0%, *p* < 0.001). However, there were no significant intergroup differences in frequency of recurrence in the lungs, distant lymph nodes, and locoregional site.

**Table 5:** Comparison of recurrence sites between ER and non-ER groups

Recurrence site	ER group	Non-ER group	*p*
Liver	26 (57.8%)	22 (16.5%)	< 0.001
Lung	2 (4.4%)	19 (14.3%)	0.08
Peritoneum	14 (31.1%)	15 (11.3%)	0.002
Distant lymph node	10 (22.2%)	26 (19.5%)	0.70
Locoregional site	12 (26.7%)	25 (18.8%)	0.26

*ER* early recurrence, *Non-ER* no recurrence within 6 months after resection

## Discussion

In this study, we established early postoperative recurrence prediction scores based on preoperative serum CA19-9 and SPan-1 levels, as well as preoperative tumor diameter in 178 patients with resectable PDAC who had undergone radical resection. We showed that it was possible to stratify postoperative RFS and OS depending on the predicted score. We also demonstrated that the median RFS time for patients with the prediction score of 3 (preoperative CA19-9 level above 133 U/mL, SPan-1 level above 78.2 U/mL, and CT tumor diameter above 23 mm) was 5.5 months. Furthermore, multivariate analysis revealed that a score of 3 was the independent factor associated with recurrence within 6 months after surgery. With respect to the recurrence pattern, the ER group more often had liver metastasis and peritoneal dissemination compared with the non-ER group. In patients with a prediction score of 0, the median RFS and the median OS time were 24.4 and 51.8 months, respectively, which indicated notably better prognosis. The early recurrence prediction score was able to stratify the prognosis of resectable PDAC and may be extremely useful for treatment decision-making.

The definition of early recurrence after surgery for PDAC varied among previous reports, from 6 months^30,31^ and 8 months,^32^ to 12 months^33^ after surgery. Groot et al.^18^ classified PDAC patients on the basis of the postoperative recurrence time monthly from within 3 months to within 20 months, and evaluated the difference in prognosis between early and late recurrence focusing on the *p*-value of the survival curve analysis. They showed that the cutoff value for the early recurrence time, which showed the greatest difference in prognosis, was set 12 months after surgery.^18^ They also reported that adjuvant therapy was one of the independent factors that reduced the risk of early recurrence.^18^ Postoperative adjuvant chemotherapy is generally administered for 6 months,^6,8,34,35^ and completion of adjuvant chemotherapy has been reported to be a key factor of long-term survival.^36,37^ However, if recurrence occurred within 6 months after surgery, long-term survival would not be expected because the postoperative adjuvant chemotherapy could not be completed. To identify such cases with extremely malignant tumor behavior, in this study we defined early recurrence as PDAC recurrence within 6 months after surgery.

CA19-9 antigen is a sugar chain antigen derived from cell membrane glycolipid expressed in colorectal cancer cells, which was first reported by Koprowski.^38^ Since then, many investigators have reported that the antigen was expressed not only in colorectal cancer but also in other gastrointestinal cancers such as PDAC and cholangiocarcinoma.^39,40^ In PDAC, serum CA19-9 levels have been reported to reflect the prognosis of PDAC,^11,41,42^ and the link between cutoff values of preoperative serum CA19-9 levels and early postoperative recurrence and poor prognosis was also investigated.^20,30,43–47^ On the other hand, SPan-1 is a cancer-related antigen recognized by a monoclonal antibody prepared using the human PDAC cell line SW1990 as an immune antigen, and it is a high-molecular-weight mucin-like protein.^48^ Although the sensitivity of SPan-1 for PDAC diagnosis is equivalent to that of CA19-9, SPan-1 is a distinguishing antigen that is synthesized even in Lewis-negative patients.^49^ Similar to CA19-9, SPan-1 is a tumor marker that reflects the prognosis of PDAC,^50^ and it has been reported as a risk factor for recurrence within 6 months after surgery.^44,51^ In addition, the usefulness of immunonutrient indicators NLR^10^ and mGPS^31^ as factors related to early recurrence has also been reported. Furthermore, stratification of the prognosis after curative surgery using the factors related to early recurrence has been reported in previous studies.^19,20,31,45,52,53^ In our study, the prediction scoring system using preoperative serum CA19-9, SPan-1, and tumor diameter on CT could stratify the survival time of each score. In addition, median RFS and OS in the ERP score 3 groups were estimated to be 5.5 and 15.9 months, respectively, and this group was considered the highly aggressive PDAC group.

In the present study, CA19-9, SPan-1, and the tumor diameter on CT were also identified as important factors for predicting the early postoperative recurrence of PDAC. CA19-9, SPan-1, and the tumor diameter on CT are factors that indicate the malignant potential of PDAC, and they can be assessed prior to initiating treatment. The combination of these factors may be highly significant for predicting and classifying aggressive PDAC with higher malignant potential before initiating treatment. Matsumoto et al.^31^ conducted a multicenter study and reported that CA19-9 levels, tumor diameter > 30 mm, and a modified Glasgow Prognostic Score of 2 were the independent factors associated early recurrence within 6 months after surgery in patients with R-PDAC. They also demonstrated that the combination of these factors could stratify patient prognosis. In addition, Isaji et al.^13^ proposed a modified definition and criteria for borderline resectable (BR)-PDAC, including CA19-9 > 500 U/mL, lymph node metastasis confirmed by biopsy or PET-CT, and an Eastern Cooperative Oncology Group Performance Status (ECOG-PS) > 2. Thus, evaluating malignant potential using the preoperative CA19-9 level and other preoperative factors is extremely useful for formulating a treatment plan.

CA19-9 is a tumor marker that recognizes an epitope of the sialyl Lewis A antigen.^54,55^ It is also known that DUPAN-2 recognizes the antigenic epitope of sialyl Lewis C, which is a precursor of sialyl Lewis A antigen.^54^ Conversely, some details about the epitope recognized by SPan-1 are unclear, but it is believed that the epitope is similar, but not identical, to that of CA19-9. It has also been reported that SPan-1 recognizes the CA19-9 epitope and some of the antigenic epitopes of sialyl Lewis C recognized by DUPAN-2.^49^ When the Lewis blood group is negative, the Lewis antigen gene enzyme that synthesizes the sialyl Lewis A from the sialyl Lewis C is deficient. As a result, Lewis-negative PDAC is negative for CA19-9.^56^ However, DUPAN-2 and SPan-1 are unique in that they recognize the antigenic epitope of sialyl Lewis C, which is a precursor of the antigenic epitope of sialyl Lewis A. Therefore, they can be evaluated even in patients with Lewis-negative PDAC. Regarding the evaluation of the malignant potential of PDAC, some reports found that the combination of CA19-9 and SPan-1 could be used to evaluate disease activity during chemotherapy for PDAC^57^ and that the combination of CA19-9 and DUPAN-2 could be used to estimate the prognosis of PDAC.^58^ Similarly, the preoperative tumor marker index using the cutoff values of CA19-9, CEA, DUPAN-2, and SPan-1 was useful for predicting the prognosis of PDAC.^59^ As described previously, although SPan-1 and DUPAN-2 could not be biomarkers beyond CA19-9 on their own, when combined with CA19-9, they might act in a complementary manner to enable a more sensitive classification of the malignant potential of PDAC. Although DUPAN-2 was not identified as a predictor of early recurrence in this study, it is considered an important factor in the assessment of malignant potential including early recurrence.

In addition to CA19-9 and SPan-1 levels, the tumor diameter on CT was also a significant factor predictive of early recurrence. Tumor diameter is an imaging parameter associated with major vascular invasion. As tumor size increases, the likelihood of vascular invasion such as portal vein or superior mesenteric artery invasion increases, and vascular invasion is consequently associated with potential multi-organ metastasis via blood vessels.^60^ However, in this study, portal vein resection and histological portal vein invasion were not associated with early recurrence. Therefore, tumor size might be an indicator of malignant potential, separate from vascular invasion. Tumor diameter is an imaging parameter that is not strictly a biomarker. However, the aforementioned findings suggest that tumor diameter could be a biomarker for predicting early recurrence in patients with R-PDAC. These results suggest that malignant potential can be evaluated in a complementary manner by combining multiple tumor markers and biomarkers, such as tumor size, and that early recurrence of PDAC within 6 months after surgery can be predicted more sensitively. The ability to predict patients at risk of early recurrence within 6 months before initiating treatment is considered extremely meaningful in terms of avoiding unnecessary surgery.

In our study, significantly high rates of no postoperative adjuvant chemotherapy, positive margins, and regional lymph node metastasis were observed in the ER group. These factors may indicate the malignant potential leading to ER in PDAC. However, they can be mitigated by NAT. The efficacy of postoperative adjuvant chemotherapy has been widely reported.^6,7,61^ Adjuvant chemotherapy aims to control local microscopic tumor cells and circulating tumor cells after surgery, and ultimately to improve the postoperative recurrence and survival rates.^62^ However, several problems have been pointed out, such as the inability to introduce or complete postoperative adjuvant chemotherapy owing to delayed recovery from postoperative complications.^63^ Our study also showed that no postoperative adjuvant chemotherapy tended to be associated with ER. Alternatively, NAT can effectively control tumors by introducing systemic treatment when the patient is in the best state. Therefore, for patients with high malignant potential, as indicated by the ERP score, a much longer NAT can be employed for sufficient systemic control. With regard to positive margin, this is an independent poor prognostic factor and an early recurrence-related factor in PDAC.^64,65^ Accurately identifying the extent of PDAC may be difficult, even if it is considered resectable on imaging. In such cases, resection margins are more likely to be positive; consequently, sufficient prognosis would not be provided. NAT is likely to result in tumor shrinkage, which is meaningful in preventing positive resection margins. Lastly, regional lymph node metastasis is also a poor prognostic factor in PDAC. However, the contribution of lymph node dissection to prolonged prognosis in lymph node-positive PDAC is unclear.^66–68^ If regional lymph node metastases are controlled by NAT, ER can be prevented and postoperative survival can be prolonged.

Recently, the usefulness of NAT not only for borderline resectable PDAC but also for resectable PDAC has been reported.^22,23,69^ In a multicenter randomized controlled trial, Motoi et al. compared preoperative chemotherapy with GEM and S-1 with upfront surgery (UpS) for resectable PDAC.^70^ At the ASCO-GI meeting in 2019, results of the randomized control trial (RCT) were reported. The results showed that preoperative NAT with GEM/S-1 showed a significantly higher median survival time (MST) compared with the UpS group.^71^ On the other hand, the disadvantages of NAT have also been reported.^72^ In a meta-analysis of NAT for resectable PDAC, Zhan et al. reported that NAT had no apparent effect on prolonging survival. They suggested that NAT should be carefully indicated for resectable PDAC because of the possibility of disease progression during NAT and the need for more invasive diagnostic techniques for pretreatment diagnosis.^72^ In 2020, a multicenter phase III trial comparing preoperative chemoradiotherapy (CRT) for resectable PDAC with UpS reported that CRTs for borderline resectable and resectable PDAC had no effect on survival prolongation.^73^ They investigated the usefulness of preoperative chemoradiotherapy (CRT) for resectable and BR-PDAC. In the intention-to-treat analysis, the preoperative CRT group had significantly better disease-free survival and locoregional failure-free survival; however, there was no significant difference in OS between the two groups. Furthermore, subgroup analysis did not reveal a contribution of preoperative CRT to OS in patients with R-PDAC. However, in this study, the chemotherapy used during preoperative CRT was gemcitabine, which was not as potent as gemcitabine/nab-paclitaxel or FOFIRINOX. Further clinical studies on preoperative CRT for R-PDAC with such agents are expected to clarify this issue.

Concerning NAT for R-PDAC, it remains controversial because there was not sufficient evidence to demonstrate the prognostic advantages. One of the reasons is that, even if the PDAC was considered to be resectable based on the CT image, it includes a group of cases with an extremely poor prognosis that recur within 6 months after the radical resection. In our study, in PDAC classified as resectable on the CT images, we found that liver metastasis and peritoneal dissemination were significantly more common in the group that recurred within 6 months after radical resection. It is possible that such groups may have potentially distant metastases at the time of surgery, or may have extremely high malignancy that cannot be controlled by surgery. Even if several courses of NAT were introduced in these groups with extremely poor prognosis, it would be difficult to control such tumor dynamics and prolong postoperative survival. For such PDACs with high malignant potential, a long-term NAT for sufficient tumor control might be indicated to improve the survivals.

Several reports have been published on the prediction of ER of PDAC and treatment selection based on the prediction. Oba et al.^74^ defined radiologically occult metastatic pancreatic cancer (ROMPC) as distant metastases revealed during surgery or recurrence within 6 months after surgery. They stated that ROMPC had a significantly lower survival rate than non-ROMPC, and upfront surgery for ROPMC is not beneficial. They also reported that the risk of ROMPC can be predicted if both CA19-9 level of > 300 U/mL and tumor diameter of > 30 mm were met. They suggested that ROMPC should be predicted preoperatively to apply neoadjuvant chemotherapy. Kurahara et al.^45^ studied the ER of radiographically R-PDAC. They reported that CA19-9 level of > 85 U/mL and a p53 expression rate of 0% or > 80% in tumor cells were independent risk factors for ER. Patients who presented at least one of these factors were defined as high risk, and their survival rates were compared with those of NAC patients. NAC had a survival benefit in the high-risk group. Takahashi et al.^75^ classified radiographically R-PDAC with CA19-9 levels of > 120 U/mL as biologically BR-PDAC (bBR-PDAC). Although the survival rate of bBR-PDAC was comparable to that of anatomically BR-PDAC, the prognosis was significantly improved when CA19-9 was normalized by preoperative CRT. However, in the same report, they found that CA19-9 levels did not normalize after preoperative CRT in approximately 50% of cases, which were associated with a significantly higher rate of distant recurrences. Thus, predicting early recurrence by using preoperative factors such as CA19-9 and applying NAT may improve the prognosis of the early recurrence group. However, further clinical studies with a higher level of evidence should be conducted.

In our study, the median RFS was only 5.5 months for individuals with an early recurrence prediction score of 3 points (e.g., with CA19-9 > 133 U/mL, SPan-1 > 78.2 U/mL, and CT tumor diameter > 23 mm). Furthermore, their median OS was 15.9 months, which was an extremely poor prognosis in spite of R-PDAC. Such poor prognosis groups could be considered biologically unresectable PDAC and, therefore, it seems that the treatment strategy aiming at conversion surgery^76^ after sufficient tumor control by chemotherapy or total neoadjuvant therapy (TNT)^77^ should be applied. TNT is a reported preoperative treatment for borderline resectable and locally advanced unresectable PDAC that combines preoperative intensive chemotherapy and chemoradiotherapy. The treatment concept of TNT is combining systemic control for occult micro-metastasis with local control for negative resection margins. Truty et al.^77^ have reported the usefulness of TNT for BR/LA PDAC, suggesting that long-term chemotherapy of more than six cycles, negative CA19-9 after chemotherapy, and sufficient pathological response were independent factors of long-term survival. Although TNT is a therapeutic strategy for BR/LA PDAC, it should be indicated in cases with resectable PDAC that are biologically unresectable PDAC, such as those with an early recurrence prediction score of 3. Therefore, the ERP scoring system may have great significance in identifying patients with R-PDAC and poor prognoses and avoiding unnecessary surgery.

On the other hand, the group classified with ERP score of 0 had a median RFS of 24.4 months, a median OS of 54.8 months, and a 5-year survival rate of 49.2%, which was a notably better prognosis. According to the previous reports^71,78–80^ that investigated the efficacy of NAT for resectable PDAC, MST of NAT group was 17.4–38.2 months, compared with MST in surgery-first group of 14.4–26.4 months. The recent report by Unno et al.,^71^ which was a multicenter randomized controlled trial comparing NAT with GEM and S-1 with UpS for resectable PDAC, also reported that MST of NAT group was estimated to 36.7 months (HR, 0.72; *p* = 0.015). Judging from the long MST of 54.8 months of the prediction score 0 in our study, the prognosis of the group would not be expected to be further improved by NAT. Instead, appropriate upfront surgery and the prompt introduction of adjuvant chemotherapy should be indicated for such a group. Recently, much better prognosis has been reported with adjuvant chemotherapy with FOLFIRINOX.^8^ Therefore, for the group classified as our ERP score of 0, postoperative complications and malnutrition after radical surgery should be prevented as much as possible, and postoperative adjuvant chemotherapy should be smoothly introduced according to the previous reports.^37,81^

This study had the following limitations. First, this was a retrospective study with a limited number of patients in a single institution. Second, since 5–10% of Japanese have Lewis-negative blood type, CA19-9 could not be detected in such cases. However, Lewis blood type was not assessed in this study. Third, postoperative adjuvant chemotherapy was not unified, and multiple regimens were used. Fourth, due to the historical background, there were cases in which efficacious chemotherapy for PDAC such as GEM and nab-paclitaxel combination therapy or FOLFIRINOX could not be used for recurrence treatment, and there might be a bias in survival time after recurrence. Fifth, our cases included obstructive jaundice cases, which may have affected the levels of CA19-9 and SPan-1. Sixth, PET-CT and cytology with diagnostic laparoscopy for preoperative distant metastasis evaluation were not performed in all patients. Seventh, our study did not address the optimal method or duration of NAT to control tumor dynamics. When NAT has no obvious effect on patients with high ERP scores, in reality, radical surgery often becomes the only option. Thus, preoperative stratification of prognosis using ERP scores may not be practical in determining the treatment strategy. Finally, in our study, cases with R1 resection were also included. In principle, cancer relapse after R1 resection might be considered progression and not recurrence. The ER group may thus have included two different tumor dynamics: recurrence and progression. Despite these limitations, this study had a strong point that the early recurrence prediction scoring system could estimate an ER group with a poor prognosis, which could help to avoid meaningless surgery.

In conclusion, for resectable PDAC, an early recurrence prediction scoring system using preoperative serum CA19-9 level, preoperative serum SPan-1 level, and preoperative CT tumor diameter was effective to predict early recurrence within 6 months after surgery. The system could have great significance in avoiding unnecessary surgery. Furthermore, it could stratify the patients according to their predictive prognosis, which may be useful for determining the adaptation to neoadjuvant therapy for resectable PDAC.
